# Composable free-space continuous-variable quantum key distribution using discrete modulation

**DOI:** 10.1126/sciadv.adv1440

**Published:** 2026-06-12

**Authors:** Kevin Jaksch, Thomas Dirmeier, Yannick Weiser, Stefan Richter, Ömer Bayraktar, Bastian Hacker, Conrad Rößler, Imran Khan, Stefan Petscharning, Thomas Grafenauer, Michael Hentschel, Bernhard Ömer, Christoph Pacher, Florian Kanitschar, Twesh Upadhyaya, Jie Lin, Norbert Lütkenhaus, Gerd Leuchs, Christoph Marquardt

**Affiliations:** ^1^ Max Planck Institute for the Science of Light, 91058 Erlangen, Germany.; ^2^ Department of Physics, Friedrich-Alexander-Universität Erlangen-Nürnberg, 91058 Erlangen, Germany.; ^3^ SAOT, Graduate School in Advanced Optical Technologies, 91052 Erlangen, Germany.; ^4^ AIT Austrian Institute of Technology, Center for Digital Safety & Security, 1210 Vienna, Austria.; ^5^ fragmentiX Storage Solutions GmbH, 3400 Klosterneuburg, Austria.; ^6^ Vienna Center for Quantum Science and Technology (VCQ), Atominstitut, Technische Universität Wien, 1020 Vienna, Austria.; ^7^ Institute for Quantum Computing and Department of Physics and Astronomy, University of Waterloo, Waterloo, Ontario N2L 3G, Canada.

## Abstract

Continuous-variable (CV) quantum key distribution (QKD) allows for quantum secure communication with the benefit of being close to classical coherent communication. In recent years, CV QKD protocols using a discrete number of displaced coherent states have been studied intensively as the modulation can be directly implemented with real devices with finite resolution. Until now, experiments only calculated key rates in the asymptotic regime. Here, we present a CV QKD system using discrete modulation that is especially designed for atmospheric channels. We use polarization encoding to exploit the nonbirefringent nature of the turbulent atmosphere. This allows to expand CV QKD networks beyond the existing fiber backbone. In a laboratory demonstration with a static 3-decibel loss channel, we implemented a recently developed security proof allowing to calculate composable finite-size key rates against independently and identically distributed collective attacks. We applied the full QKD protocol including a quantum random number generator, error correction, and privacy amplification to extract secret keys.

## Teaser

A polarization-encoded continuous-variable QKD system is used to generate composable secret keys against collective attacks.

## INTRODUCTION

Continuous-variable (CV) quantum key distribution (QKD) can use squeezed or coherent states (*1*–*3*) and homodyne detection to allow for quantum secure communication between two parties while being close to existing classical coherent communication (*4*–*6*). For long, research focused on Gaussian-modulated protocols, where coherent states are displaced according to a two-dimensional (2D) Gaussian distribution. This allowed to exploit the optimality of Gaussian attacks and to simplify the security proof techniques (*7*). This lead to tremendous advances in fiber-based systems, demonstrating long-distance CV QKD (*8*, *9*) even for true local oscillators (LOs) (*10*) and embedding the systems in composable frameworks against collective attacks for both practical block sizes (*11*) and resistant against modulation imperfections (*12*).

However, the continuous Gaussian distribution remains a theoretical idealization as experimental implementations are limited by the finite discretization of the used electronic devices. Thus, discrete-modulated (DM) systems with a finite number of displaced coherent states have been investigated (*13*–*15*). Yet, one cannot assume the optimality of Gaussian attacks for discrete modulation. Without that, the security proofs had the challenge of working in the infinite dimensional Hilbert space populated by coherent states.

In recent years, new proof techniques have been able to overcome this limitation. For example, for asymptotic security proofs (*16*–*18*), there have been several experimental demonstrations using quadrature amplitude modulation (QAM) with 16 to 256 displaced states (*19*–*21*). Here, no error correction was implemented yet and the authors hence only gave estimates on the achievable asymptotic key rates assuming error correction with an efficiency of β=95% with respect to the Shannon limit of the classical channel between Alice and Bob. Another experiment also demonstrated a four-state quadrature phase-shift keying (QPSK) protocol with implemented low-density parity-check (LDPC) codes for error correction (*22*). They, however, did not study the impact of the error correction on the real extractable key but rather used the measured efficiencies of the LDPC codes to calculate asymptotic key rates again. In general, it occurs that many experimental papers in CV QKD [e.g., (*23*, *24*)] deal with error correction by assuming a certain efficiency β and adding the potential frame error rate (FER) as a scaling factor to the asymptotic key rate formula $K=\left(1-\text{FER}\right)\cdot \left(\beta I_{\text{AB}}-\chi_{\text{BE}}\right)$. This leads to the conclusion that higher key rates are obtained when operating the system close to the threshold of the used error correction code (ECC), leading to a high efficiency while increasing the FER. This approach has already raised concerns in recent years (*25*).

Another proof technique for asymptotic DMCV QKD using a numerical approach (*26*, *27*) was introduced in (*28*). This approach has been proven to be very versatile, being able to include a trusted detector model (*29*, *30*) and even composability against independently and identically distributed (i.i.d.) collective attacks (*31*). Yet, an experimental implementation of this kind of protocols is missing and will be presented in this paper. Another theoretical approach for composable security against i.i.d. collective attacks was shown in (*32*). Very recently, new proofs have made partial progress toward proving security under coherent attacks (*33*–*35*), another paper generalized the work of (*29*, *30*) to the multiuser scenario (*36*).

All of the mentioned work so far was done for fiber-based systems, which can take advantage of the existing infrastructure in metropolitan areas. Complementary, free-space systems will allow to expand QKD networks beyond this existing but fixed fiber backbone. Here, the feasibility for CV QKD for both satellite-to-ground (*37*–*39*) and terrestrial urban links (*14*, *40*, *41*), as well as the general treatment of fading channels (*42*–*44*), has been studied intensively in recent years. With a full atmospheric implementation still missing, several laboratory studies to investigate technical aspects like rate-adaptive error correction (*45*) or the effect of turbulences on wavefront distortions (*46*) show the vast interest in free-space applications.

Besides amplitude and phase encoding, which is used in fiber-based CV QKD systems, polarization encoding has proven to be an advantageous degree of freedom in a turbulent but nonbirefringent atmosphere. Several studies showed the distribution of polarization coherent (*14*) and squeezed states (*47*) over an urban atmospheric link. Another experiment showed the feasibility of an unidimensional Gaussian-modulated protocol in the asymptotic regime (*40*). However, polarization-based systems did not yet demonstrate a full QKD implementation to generate secret keys or were applied to advanced security proofs including finite-size effects or composability.

In this work, we merge these recent endeavors in free space and DMCV QKD. We developed a mobile QKD system that uses discrete QPSK modulation in the Stokes parameters. Using polarization modulation and Stokes detection allows for quantum communication in turbulent but nonbirefringent atmospheric channels and is equivalent to homodyne detection using a transmitted LO (*48*). In our system, we also implemented the full QKD pipeline including a quantum random number generator (QRNG), error correction, and privacy amplification. In a laboratory demonstration, we applied the security statement of (*31*) to calculate composable finite-size key rates with a total security parameter of $\epsilon =1\times 10^{-10}$ against i.i.d. collective attacks. We further applied the postprocessing pipeline to generate secret keys and studied the effect of frame errors during error correction. For this, we do not only add a scaling factor to the key rate but also stay in the framework of the given security statement. This shows that the generated key length is strongly dependent on the FER.

Simultaneously to this paper, composable security for DMCV QKD was also experimentally demonstrated for phase-encoded systems designed for fiber channels (*49*). During the review process, further experiments expanded this composable framework to integrated photonic chips (*50*) and 16QAM modulation (*51*).

## RESULTS

### Polarization-encoded modulation

As the system is designed for atmospheric quantum communication, we exploit the nonbirefringent nature of the atmosphere and use the polarization degree of freedom to encode the quantum states (*14*). This is different from amplitude and phase encoding, which is used in fiber-based systems and is subject to fluctuations in atmospheric turbulences.

Despite using a different physical degree of freedom, those two modulation schemes can be seen as equivalent, given that the LO is not prepared locally at the receiver but sent through the quantum channel (transmitted LO scenario). As described in this chapter, the measurement of both schemes is then described with the means of two-mode Stokes operators, corresponding to the measurement of the single-mode quadrature operators.

We prepare the bright LO and the coherent signal states in the same spatial mode but in orthogonal circular [left-handed (L) and right-handed (R)] polarization modes (*52*)

$$|\psi \rangle =|\alpha_{\text{LO}}\rangle_{L}|\alpha_{n}\rangle_{R}$$ (1)

with a discrete QPSK modulation for the signal states $\alpha_{n}=|\alpha |\cdot \text{exp}\left[\frac{in\pi }{2}\right]$, n∈{0,1,2,3}, and $|\alpha_{\text{LO}}|\gg |\alpha |$.

The polarization can thus be described using Stokes operators (*52*)

$$\begin{array}{cc} {\hat{S}}_{0}={\hat{a}}_{L}^{†}{\hat{a}}_{L}+{\hat{a}}_{R}^{†}{\hat{a}}_{R}, & {\hat{S}}_{1}={\hat{a}}_{L}^{†}{\hat{a}}_{R}+{\hat{a}}_{R}^{†}{\hat{a}}_{L}\\ {\hat{S}}_{2}=i\left({\hat{a}}_{R}^{†}{\hat{a}}_{L}-{\hat{a}}_{L}^{†}{\hat{a}}_{R}\right), & {\hat{S}}_{3}={\hat{a}}_{L}^{†}{\hat{a}}_{L}-{\hat{a}}_{R}^{†}{\hat{a}}_{R} \end{array}$$ (2)

where we express the mode operators in the circular basis with ${\hat{\alpha }}_{R/L}=\frac{1}{\sqrt{2}}\left({\hat{a}}_{x}\pm i{\hat{a}}_{y}\right)$. As discussed in (*48*), the two-mode picture of Stokes operators is also the correct way to describe homodyne detection to measure the quadrature operators $\hat{X}/\hat{P}$ in a phase-encoded system for an LO transmitted through the untrusted optical channel. For that, one has to interchange the two orthogonal polarization modes with the two separate spatial modes of the LO and the signal beam. For the given scenario of a bright L-polarized LO ${\hat{a}}_{L}\to |\alpha_{L}|\text{exp}\left(i\phi \right)$, this means that the Stokes detection of ${\hat{S}}_{1}$ and ${\hat{S}}_{2}$ corresponds to a quadrature measurement of ${\hat{X}}_{R}$ and ${\hat{P}}_{R}$ in the signal’s orthogonal, R-polarized mode (*53*)

$$\begin{array}{c} {\hat{S}}_{1}\approx |\alpha_{L}|\left({\hat{a}}_{R}^{†}+{\hat{a}}_{R}\right)=\sqrt{2}|\alpha_{L}|{\hat{X}}_{R}\\ {\hat{S}}_{2}\approx i|\alpha_{L}|\left({\hat{a}}_{R}^{†}-{\hat{a}}_{R}\right)=\sqrt{2}|\alpha_{L}|{\hat{P}}_{R} \end{array}$$ (3)

The relative phase ϕ is hereby set to zero because both polarization modes have no relative phase shift. For the last step, we use natural units and define the quadrature operators by $\hat{X}=\frac{1}{\sqrt{2}}\left({\hat{a}}^{†}+\hat{a}\right)$ and $\hat{P}=\frac{i}{\sqrt{2}}\left({\hat{a}}^{†}-\hat{a}\right)$. We use this normalization throughout this work, e.g., when transforming between amplitude and quadrature variables with $x=\sqrt{2}ℜ\left(\alpha \right)$ and $p=\sqrt{2}ℑ\left(\alpha \right)$.

Please note that the used approximation is the standard one for homodyne detection in the bright LO limit (*54*). The corresponding uncertainty relation is given by

$$\text{Var}\left({\hat{S}}_{1}\right)\cdot \text{Var}\left({\hat{S}}_{2}\right)\geq {\left|\langle {\hat{S}}_{3}\rangle \right|}^{2}$$ (4)

being equal for coherent polarization states and increased in the presence of polarization excess noise. Having both the LO and the signal mode share the same spatial mode has another advantage for atmospheric applications: Both modes are getting distorted by turbulences, leading to phase fluctuations in the LO and the signal mode. However, because both modes are traveling in the same spatial mode, those phase distortions are the same. Thus, there is no phase drift ϕ between the LO and the signal in the balanced homodyne detection picture of our measurement (Eq. 3), meaning that the phase space is not rotating over time. The stability of polarization encoding for quantum communication in turbulent environments was demonstrated over several urban atmospheric channels (*14*, *40*, *47*, *55*, *56*).

### Experimental implementation

**Fig. 1.**
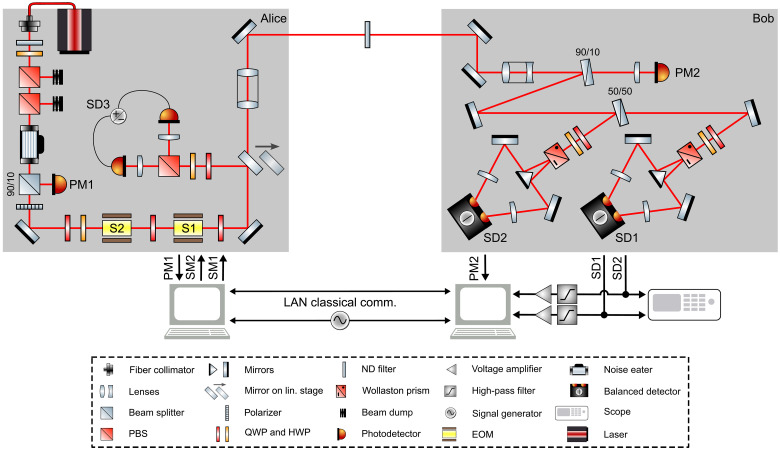
Experimental setup. At the sender, a circularly polarized bright LO with a discrete QPSK modulation in the Stokes $S_{1}$ and $S_{2}$ parameters is prepared. At the receiver, the signal is measured by direct detection to monitor the transmission and two identical Stokes detection units, analogous to a heterodyne measurement in the optical phase space. The atmospheric channel is emulated by a 3-dB ND filter. Used abbreviations: PBS, polarizing beam splitter; QWP/HWP, quarter-wave plate/half-wave plate; ND filter, neutral density filter; EOM, electro-optical modulator; SM, Stokes modulation; SD, Stokes detection; PM, power monitoring.

The experimental setup consists of a breadboard-mounted and mobile sender (Alice) and receiver (Bob) module and is suitable for a flexible deployment in atmospheric links (see Fig. 1). The light of a continuous wave laser at 810 nm is spatially mode cleaned by a polarization-maintaining fiber and collimated into free space. A stack of neutral density (ND) filters, a half-wave plate and polarizing beam splitters are used for coarse and fine power adjustment before the power is stabilized with a noise eater. A part of the beam is used to monitor the power on the sender side, the main fraction is sent through a combination of multiple wave plates and polarization modulators. Our system uses free-space electro-optical modulators (EOMs; Thorlabs model EO-AM-NR-C1) with a bandwidth of ~100 MHz, driven with modulation voltages below 5 V_pp_, which is sufficient for our purposes.

By that combination, we prepare a circularly L-polarized strong LO with an additional discrete QPSK modulation of four coherent states in the orthogonal R-polarization mode. The signal at Alice can be measured with a first Stokes detection unit with two identical but separately amplified PIN photodiodes, labeled SD3 in Fig. 1, to quantify the laser’s intensity noise by comparing the sum and difference signal. For this laboratory demonstration, we replaced an atmospheric channel with a short-distance free-space channel and a 3-dB ND filter.

On Bob’s side, a fraction of the beam is sent to a second monitoring detector to determine the transmission of the system. The remainder of the beam is split equally to two identical Stokes detection units to measure the Stokes ${\hat{S}}_{1}$ and ${\hat{S}}_{2}$ operator. In contrast to Alice’s side, Bob uses balanced detectors that allow us to suppress correlated classical noise on the optical signal before amplification and further processing. The dc-coupled output of the receiver is split and one part is monitored by a scope to check for imbalancing of the detectors. The rest of the signal is, at first, high-pass filtered at 130 kHz to counteract low-frequency drifts and then voltage amplified to match the dynamic range of the used analog-to-digital converter (ADC). As further discussed in Materials and Methods, the radio frequency (rf) spectra of the quantum signals is shifted away from the low-frequency regime to avoid interference with the filter operation. As a countermeasure for hacking attacks on the transmitted LO, we foresee to add a local vacuum calibration at random times and a Stokes ${\hat{S}}_{3}$ detection (*48*) at Bob.

Clock synchronization between the sender and the receiver is achieved by a shared 10-MHz signal from a function generator, which can be exchanged by two free-drifting Rubidium clocks at the sender and the receiver, respectively, for future applications with separate locations. The classical communication between Alice and Bob is achieved via a LAN Ethernet connection.

### Signal preparation

**Fig. 2.**
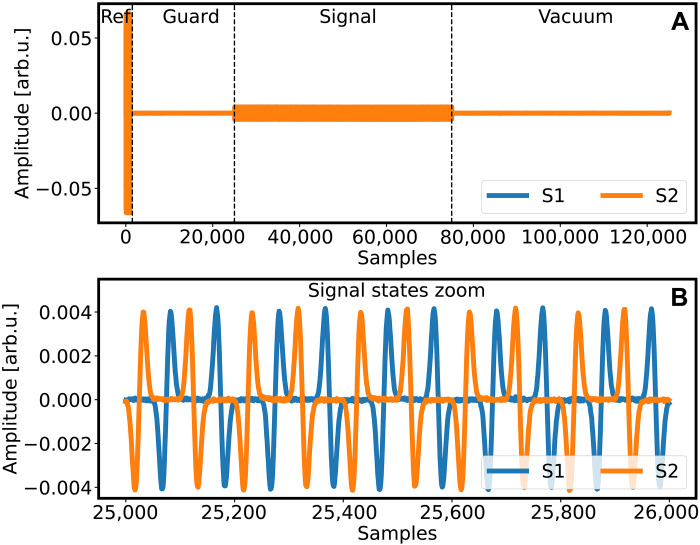
Measured modulation pattern in the electrical domain as sent from the digital-to-analog (DAC) converter. The sampling rate is $f_{\text{DAC}}=2$ GHz. (**A**) Time-multiplexed frame with four slots for reference, guard, signal and vacuum states. arb.u., arbitrary units. (**B**) Zoom into the signal states, encoded in Hermite-Gaussian pulses. For clarity, the plot is averaged over 240 frames with the same signal pattern.

**Fig. 3.**
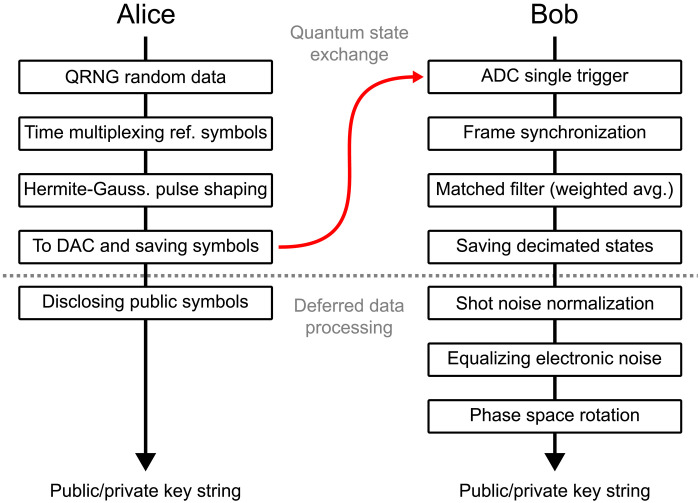
Sketch of the DSP routine. (**Left**) Alice’s steps used during signal preparation. (**Right**) Bob’s steps used during signal processing. The resulting public key strings are used for energy and acceptance testing. The resulting private key strings are fed into the AIT-QPS pipeline.

The system is operated in a burst mode, and its modulation pattern is designed to meet the requirements of future applications in atmospheric links. In a burst, Alice prepares 240 time-multiplexed frames each consisting of 2500 states modulated with 25 MHz, as shown in Fig. 2. Each state is then defined on a 40-ns pulse window and temporally encoded in oversampled Hermite-Gaussian pulses with different polarity and amplitudes (see Materials and Methods). The first 20 states are bright reference states with a fixed pattern used for triggering the ADC and frame synchronization. As the modulators may endure ringing or thermal effects after being driven by higher voltages, the next 480 states are neglected to avoid influence on the following 1000 signal states. For the signal, a random key string is provided by presaved random numbers from a fiber-integrated QRNG, which is based on the balanced homodyne detection of the optical vacuum state (*57*). The entropy estimation was performed using a model considering a classical adversary (*58*). Two-universal hashing (*59*) by means of the Toeplitz matrix method (*60*) was used to obtain random numbers with a statistical distance $\epsilon_{Q}<2^{-100}$ to a uniform distribution. By using QPSK modulation, we can encode 2 bits of information in each sent state (symbol). Last, the modulation is turned off and the next 1000 states are used as a vacuum reference. Sending signal and vacuum states in the same frame copes with the fact that shot noise normalization is dependent on the power of the LO that will be sent through a fluctuating channel in atmospheric scenarios. As atmospheric turbulences are in the kilohertz regime (*61*), we define a cutoff with a safety margin at 10 kHz, so that the LO power in a 100-μs frame remains stable. This allows us to attribute a single transmission value to all the states of a frame, simplifying transmission monitoring and binning (*42*). After each burst is sent, Alice saves her key symbols for further offline processing. The digital signal processing (DSP) routine is illustrated in Fig. 3.

### Signal processing

After Bob’s ADC is triggered, the measured trace is cross-correlated with the known pattern of the reference states to properly align the frames and pulse windows. As there are no phase fluctuations between the LO and the signal mode in the polarization-based system, no additional phase correction step like in phase-encoded systems is necessary. The individual quadrature values are then recovered from the oversampled trace by calculating the weighted average over each pulse window and saved for further offline processing. Here, the weights are defined by the outgoing optical Hermite-Gaussian mode at the sender (see Materials and Methods), which was characterized before executing the QKD protocol. Like this, we ensure that the same temporal mode defining the quantum state is used for the sender and the receiver. The same weighting procedure is performed for the signal, vacuum, and detector states to have them defined according to the same temporal mode. The detector noise is measured in advance by blocking the laser beam at the receiver in the framework of a trusted detector assumption.

**Fig. 4.**
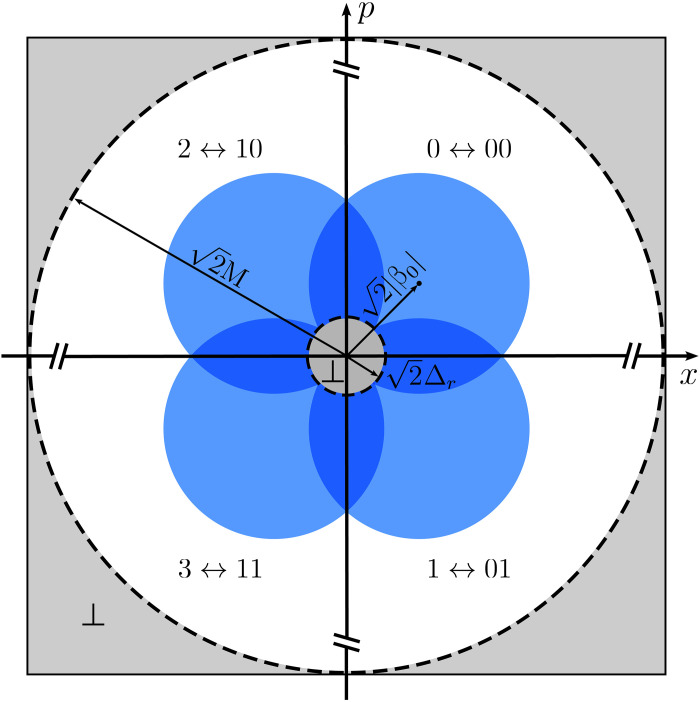
Sketch of the key map in the *x*-*p*–phase space. The blue circles illustrate the uncertainty areas of the received states with an amplitude $\beta_{n}=\sqrt{\mathrm{T\eta}}\alpha_{n}$. The uncertainty areas are defined by the standard deviation σ=1 of the *Q*-function of the coherent states, which is measured during heterodyne detection. For illustrative purposes, the uncertainty areas are drawn disproportionately large. Each measurement outcome is either discarded (⊥) or mapped to a 2-bit value, defined by the projection on the axes. This maps the QPSK pattern to two BSCs on which error correction is performed. The security proof includes the possibility of radial postselection to enhance the performance. For this experiment, we chose $\Delta_{r}=0$ and no postselection. Furthermore, detection events outside a bounded detection range M=5 are discarded. $\beta_{0}$, $\Delta_{r}$, and *M* are defined in the α-phase space [see ref. (*31*)], leading to a scaling of $\sqrt{2}$ for their representation in the *x*-*p*–phase space.

In following DSP steps, Alice uses further QRNG random numbers and announces 25% of the sent key string. For shot noise normalization, we calculate the conversion factor $\phi =\text{Var}\left(x_{\text{vac}}^{\text{meas}}\right)-\text{Var}\left(x_{\text{det}}^{\text{meas}}\right)$ and normalize the signal, vacuum, and detector traces by $x=x^{\text{meas}}/\sqrt{\phi }$. The superscript “meas” denotes measured units in volts. This is done for both measured quadratures $x^{\text{meas}}$ and $p^{\text{meas}}$ individually, which also corrects for gain differences in the two Stokes detectors. Doing so, we normalize the *Q*-function of a vacuum state to the variance $\sigma^{2}=1$ in the *x*-*p*–phase space and natural units, which includes the noise penalty of the vacuum mode that enters at the additional beam splitter during heterodyne detection (*54*). We then equalize the electronic noise of both detectors by digitally adding white Gaussian noise to the measured traces of the better detector with the lower detector noise. Adding such noise is a conservative step, making the performance of the system worse. It allows to us to work with the simplified trusted detector model of (*31*), where both detectors have the same electronic noise. We then rotate the phase space by 45°, so that the experimentally implemented on-axis modulation is rotated to the diagonals of the phase space. Whereas the on-axis modulation simplifies the experimental implementation, the bit assignment in the AIT QKD processing software (AIT-QPS) pipeline is based on a sign decision (see Fig. 4). By equalizing the electronic noise, we also prevent that different electronic noise contributions couple with each other when being rotated, which can distort excess noise calculations.

After having calculated the secret key rates, the private key strings are fed into the AIT-QPS pipeline for error correction and secret key extraction. For this demonstration, the quantum state exchange and the AIT-QPS pipeline were executed on separate computers using the LAN connection between Alice and Bob to emulate distant locations. Further offline processing steps and the key rate calculation were not yet performed locally.

### Security proof

**Fig. 5.**
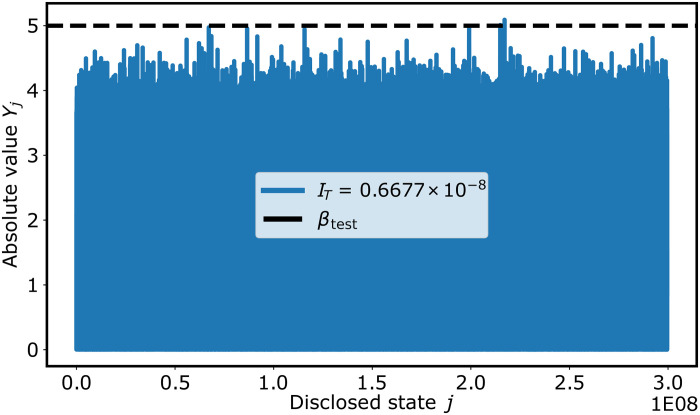
Energy test for run 3. It assures that only a small number of outliers $l_{T}^{\text{meas}}$ has an amplitude greater than $\beta_{\text{test}}=5$. With the chosen settings $n_{c}=20$ and $w=O\;\left(10^{-7}\right)$, the energy test is passed if $I_{T}=\frac{l_{T}^{\text{meas}}}{k_{T}}<1\times 10^{-8}$, allowing for maximal two outliers on the chosen disclosed block size $k_{T}$.

We discuss the underlying security argument along with the involved protocol steps and give application details for the security proof method of ref. (*31*) used in this work. After Alice has prepared and distributed *N* rounds of quantum states and Bob has performed his measurements, they proceed with statistical testing. In more detail, this means Alice and Bob disclose a random subset of size $k_{T}$ of their measurement outcomes publicly and use this information to perform an energy test and an acceptance test [see refs. (*31*, *62*) for details]. The purpose of the energy test is the following: Coherent states, as prepared in the present protocol, consist of a linear combination of all photon number states. Thus, in particular, there is no maximal photon number that would allow us to represent them in a finite-dimensional space, making the underlying key rate finding problem amenable to numerical convex optimization algorithms. Therefore, we perform an energy test that allows us, based on experimental observations, to determine the weight of our quantum states outside a finite-dimensional cutoff space of dimension $n_{c}+1$. This weight then can be used to relate the actual infinite-dimensional optimization problem to a finite-dimensional cutoff formulation of the same problem, at the cost of introducing a small weight-dependent correction (*30*). Practically, the test requires defining beforehand a cutoff dimension $n_{c}$, a testing parameter $\beta_{\text{test}}$, and a target weight *w* (see Fig. 5). We further define the number of allowed outliers $l_{T}$, where the measured amplitude is greater than $\beta_{\text{test}}$. To be independent of the public set size, this is expressed by the allowed relative number of outliers $I_{T}=\frac{l_{T}}{k_{T}}$. The target weight w∈[0,1] quantifies “how much” of Bob’s quantum state maximally may lie outside the chosen finite-dimensional cutoff space, w≥Tr[ρ(1−Π)], where Π denotes the projector on the finite-dimensional cutoff space of dimension $n_{c}+1$. Then, it counts the number of test rounds whose absolute value of the amplitude $Y_{j}=|\frac{1}{\sqrt{2}}(x_{j}+{\mathrm{ip}}_{j})|$ exceeds $\beta_{\text{test}}$, $l_{T}^{\text{meas}}=\#\left\{Y_{j}:Y_{j}\geq \beta_{\text{test}}\right\}$. $x_{j}$ and $p_{j}$ are the simultaneously measured quadratures of the *j*th state in the public set. If this number is larger than the number of allowed outliers $l_{T}$, the test fails, except with probability $\epsilon_{\text{ET}}$, and the protocol aborts. Otherwise, we proceed with the acceptance test. During the acceptance testing procedure, we compare the observed averages of certain observables with a predefined set of accepted statistics. On the basis of the expected behavior of the QKD system, the acceptance set $S^{\text{AT}}$ corresponding to the set of accepted statistics contains all density operators that could have produced the expected statistics, except with probability $\epsilon_{\text{AT}}$. Thus, practically, the sole task during the acceptance test is comparing whether the observed averages lie within the set of accepted statistics and aborting otherwise. We want to highlight that the type of security argument used in this work does not rely on any assumptions about the quantum channel connecting Alice and Bob. In contrast, the security argument is based solely on the concept of testing based on Bob’s measurement results without requiring any symmetry assumptions and rigorously handles infinite-dimensional quantum systems inherent to CV protocols, leaving merely the restriction to i.i.d. collective attacks.

On the protocol level, we proceed with a (reverse reconciliation) key map, where Bob maps his measurement outcomes (which are complex numbers in the phase space) to the set {0,1,2,3,⊥}, where ⊥ stands for symbols discarded by postselection (*3*, *63*). Intuitively, this procedure is illustrated in Fig. 4. This is followed by error correction, where Alice and Bob use the classical channel to reconcile their raw keys and error verification, as well as privacy amplification, which helps them to decouple their key from Eve’s information. Those steps are described in more detail in the next chapter and introduce the security parameters $\epsilon_{\text{EC}}$ and $\epsilon_{\text{PA}}$.

From a security proof perspective, we now have all the ingredients ready to formulate the security statement (*31*). For $\epsilon_{\text{ET}},\epsilon_{\text{AT}},\bar{\epsilon },\epsilon_{\text{EC}},\epsilon_{\text{PA}}>0$, the implemented QKD protocol is $\epsilon_{\text{EC}}+\text{max}\left\{\frac{1}{2}\epsilon_{\text{PA}}+\bar{\epsilon },\epsilon_{\text{ET}}+\epsilon_{\text{AT}}\right\}$ secure against i.i.d. collective attacks, given that, in case the protocol does not abort, the secure key length is chosen to satisfy

$$\frac{l}{N}\leq \frac{n}{N}\left[\underset{\rho \in S^{E\&A}}{\text{min}}H{\left(X|E\right)}_{\rho }-\delta \left(\bar{\epsilon }\right)-\Delta \left(w\right)-2\delta_{\text{leak}}\right]-\frac{2}{N}{\text{log}}_{2}\left(\frac{1}{\epsilon_{\text{PA}}}\right)$$ (5)

with $\delta_{\text{leak}}=\frac{{\text{leak}}_{\text{EC}}}{2n}$, where ${\text{leak}}_{\text{EC}}$ is the total number of disclosed bits, $\Delta \left(w\right)≔\sqrt{w}{\text{log}}_{2}\left(|Z|\right)+\left(1+\sqrt{w}\right)h\left(\frac{\sqrt{w}}{1+\sqrt{w}}\right),\delta \left(\epsilon \right)≔2{\text{log}}_{2}\left(\text{rank}\left(\rho_{X}\right)+3\right)\sqrt{\frac{{\text{log}}_{2}\left(2/\epsilon \right)}{n}}$, and $S^{E\&A}$ is the set of states that pass both the energy test and the acceptance test. $\bar{\epsilon }$ is the smoothing parameter used to define the smoothed version of the min entropy in (*31*), which is a “virtual” parameter we can, in principle, optimize over. By ∣Z∣, we denote the dimension of Bob’s classical system, which, in this work, is equal to 4, and by $\rho_{X}$, we denote Alice’s reduced density matrix after protocol execution.

 While the error correction leakage and the privacy amplification correction term are quantities determined by the respective software modules used, the first three terms in the key rate formula are obtained by the security argument. Because the weight was already fixed during the energy test and $\delta \left(\bar{\epsilon }\right)$ is fixed upon choosing $\bar{\epsilon }$, it remains to solve the finite-dimensional optimization problem ${\text{min}}_{\rho \in S^{E\&A}}H{\left(X|E\right)}_{\rho }$. The optimization over the set $S^{E\&A}$ can be expressed as a nonlinear semidefinite program (SDP), constrained by experimental observations along with further constraints ensuring that we are optimizing over quantum states compatible with the protocol (see the Supplementary Materials for details). In particular, we chose as the observables the displaced photon number ${\hat{n}}_{\beta_{j}}$ and the displaced squared photon number ${\hat{n}}_{\beta_{j}}^{2}$ for j∈{0,1,2,3} (see the Supplementary Materials for details), which can be derived directly from Bob’s heterodyne measurement results and are linked to the experimentally well-known quantities channel loss η and excess noise ξ. The intuition behind this choice is the following: In case of no channel noise and that the displacement was chosen in accordance with the channel loss, the expectation for those observables is zero, whereas its deviation from zero is closely linked to the excess noise. The analogous “displaced photon number basis” requires the least amount of basis states to describe the underlying quantum state up to fixed weight *w*, which makes it the natural choice.

This convex optimization problem can be solved via a method introduced in refs. (*26*, *27*) and applied to DM CV-QKD first in (*28*). It involves a two-step process, wherein the first step, a linearized version of the problem, is solved iteratively using the Frank-Wolfe algorithm (*64*). As it is not guaranteed to reach the exact minimum, the result of step 1 is merely an upper bound. However, it can serve as a starting point for step 2, where duality theory of semidefinite programming and another linearization are combined to turn this upper bound into a reliable lower bound. A relaxation takes the effect of numerical constraint violations on the key rate into account. We used CVX (*65*, *66*) to model the occurring SDPs as well as the MOSEK solver (version 10.0.34) (*67*) to solve them while coding in Matlab (version 2022a). In addition, we applied the techniques from (*29*), properly modeling and considering realistic, trusted detectors within the frame of this security proof technique. In particular, we want to highlight that the chosen security argument works directly with the DM signals without requiring any Gaussian assumption. The security proof further considers the exact constellation of every single symbol without relying on pattern averages or similar.

Please note that the system is using a two-mode Stokes measurement, whereas the security proof is based on a single-mode quadrature measurement. The security proof itself does not rely on any assumptions about the quantum channel. However, we are using the working assumption that the transmitted LO acting as the phase reference is unchanged, i.e., without an eavesdropper acting on it (honest implementation). This allows us to apply the single-mode security proof to our two-mode measurement. To formally overcome this mismatch, an additional Stokes ${\hat{S}}_{3}$ detection is planned (*48*). In addition, one needs to expand the security proof formally to the two-mode Stokes picture.

### AIT-QPS pipeline

The postprocessing pipeline is a software consisting of several modules linked in series, where the key blocks are passed along, undergoing all required steps to distill a secure final key from the incoming raw key. An instance of the pipeline is launched at either site (Alice and Bob), running the same modules, only configured to act as the respective peer. The individual steps are described in the following.

Most modules require communication with their peer module over a classical channel to exchange control information or key-related data. This channel is assumed to be error-free and completely public (e.g., an Ethernet connection). To prevent a man-in-the-middle attack, it needs to be authenticated. To this end, each key block is equipped with an authentication context, which gets passed along with the key throughout the whole process. In particular, all messages passed between peer modules are hashed into the authentication context. For information-theoretical security, a universal hash function is used, the size of which is agreed upon beforehand. After processing of the key is concluded, a preshared secret is hashed onto the context, forming the final authentication tag. Now Alice and Bob compare their tags publicly to ensure authenticity. When the pipeline is running and the preshared secret is used up, it gets replenished by QKD keys produced by the pipeline itself. For this experiment, we restarted the pipeline for each run, always authenticating with the same initial preshared key.

At first, Bob’s measured continuous data are mapped to a 2-bit value, defined by the projection on the axes of the optical phase space (see Fig. 4), and split into multiple sub-blocks for error correction. The following error correction is based on LDPC codes, operated in the binary symmetric channel (BSC) model in a reverse reconciliation mode. An LDPC code is defined by the so-called Tanner graph, which is programmatically described by a sparse binary matrix. The multiplication of the input key block with this matrix forms the syndrome (the encoded message). In reverse reconciliation, we arbitrarily define Bob’s key as correct, thus Bob encodes his key and sends the syndrome to Alice. The decoder algorithm at Alice tries to correct her key by means of belief propagation in several iterations, ultimately matching her key to the syndrome. Each LDPC code is characterized by its code rate $R=\frac{\left(L_{\text{in}}-L_{\text{syn}}\right)}{L_{\text{in}}}$, its bit error rate (BER) threshold up to which at least 50% of the key blocks can be corrected successfully and its input block length $L_{\text{in}}$. The block length $L_{\text{in}}$ = 102,400 bits is given by the dimensions of the sparse binary matrix and is the same for all used LDPC codes. $L_{\text{syn}}$ is the syndrome length, i.e., the number of disclosed bits for each corrected block. In particular, we have used three codes with rates and thresholds *R*/th: 0.06125/0.3492 (ECC#0), 0.07/0.3382 (ECC#1), and 0.08/0.3267 (ECC#2). The maximum number of iterations, after which the decoder declares a key as failed, was set to 400.

In rare cases, a collision may occur, where different keys result in the same syndrome. In this case, the LDPC decoder will erratically declare a key as successfully corrected. To further reduce the probability of such cases, an additional step of confirmation is performed. To this end, a hash of predefined size is computed by both peers and compared. The composable finite-size security model requires that all key material is passed on to privacy amplification, so failed blocks (due to both error correction and confirmation) are not discarded but openly exchanged and marked as such.

The process of privacy amplification entails the shortening of the key to render useless any information that may have leaked to an eavesdropper, both in the optical key exchange as well as during postprocessing. It should be emphasized that, as required by the security model, before privacy amplification, all key blocks are concatenated to form the initially sized input block. The size of the secure key is determined by the analysis following the security proof, in particular taking into account the amount of disclosed information during error correction and confirmation. For successfully corrected sub-blocks, this is the syndrome size and the confirmation hash size, whereas for failed sub-blocks, it is the total contained information. The actual process of key length reduction is implemented as a family of universal hash functions, one member of which is selected randomly. We use a Toeplitz matrix algorithm as hashing function, which is implemented by a fast polynomial multiplication.

### Key rate analysis

We used the developed mobile sender and receiver QKD module in a laboratory demonstration with an emulated 3-dB loss channel and performed six measurements with a block size of $N=1.20\times 10^{9}$ and a testing ratio of $r_{\text{test}}=0.25$. Doing so, we acquired enough public states for statistically bounding the observable for the aforementioned finite-size security proof. Over all runs and symbols, the average experimental parameters of the system were given by the transmission T=0.494, receiver efficiency η=0.720, detector noise $\nu_{\text{el}}=0.135$, and mean displaced (squared) photon number $\langle {\hat{n}}_{\beta }\rangle =6.70\times 10^{-4}$ and $\langle {\hat{n}}_{\beta }^{2}\rangle =6.77\times 10^{-3}$. The measured $\langle {\hat{n}}_{\beta }\rangle$ is equivalent to an excess noise of $\xi_{\text{A}}=2.71\times 10^{-3}$, defined at the channel input. Detector and excess noise are normalized with respect to the vacuum variance. A detailed list of the measured parameters for all runs and symbols can be found in the Supplementary Materials.

Please note that the key rate calculation using the numerical SDP problem takes the individual parameters for each of the four different QPSK symbols into account and does not rely on a pattern average. This is a major advantage in comparison to other security proofs in Gaussian-modulated and DM CV QKD using analytical equations. To the best of our knowledge, all previous analytical proofs assume that the parameters for all different symbols are identical, i.e., assume a perfect symmetric preparation and measurement, and therefore only use parameters (like amplitude or excess noise) averaged over all different symbols. Obviously, this assumption is violated in realistic implementations because of technical imperfections.

Being a laboratory demonstration, we are characterizing the behavior of the QKD modules over a lossy channel without an eavesdropper acting on it. Doing so, we use the observed statistics of the six runs in this so-called honest implementation to both define the nonunique acceptance sets of the security proof and to run the QKD protocol with them. In a practical implementation, one would have to test the measured observables of the untrusted channel against the beforehand defined acceptance sets. Nonunique acceptance testing allows the security proof to be complete: Even in an honest link and with stable conditions, an experimental measurement will almost never result in the exact same statistics due to finite-size fluctuations. In such an unique acceptance scenario, the protocol would almost always abort and not generate a key. As explained in (*31*), we can increase the set of accepted statistics: For each observable *W*, we allow the measurement to deviate from the expected statistics 〈W〉 by $t_{W}=t\mu_{W}$, with $\mu_{W}$ being the variation bound of observable *X* according to the acceptance testing theorem. Increasing $t_{W}$ will not only increase the acceptance set $S^{\text{AT}}$ and decrease the key rate but also decrease the abort protocol probability for a passive Eve. Here, we fixed t=1, noting that this parameter could be optimized for the next practical implementation.

The computational time of the numerical SDP problem is thus shifted to the system’s characterization and acceptance set definition beforehand, leaving us with a fast abort/nonabort decision during the protocol run. The key rates could be further improved by using acceptance testing for variable-length security proofs, as recently proposed for discrete-variable QKD protocols with a framework open to expansion to the CV regime (*68*).

**Fig. 6.**
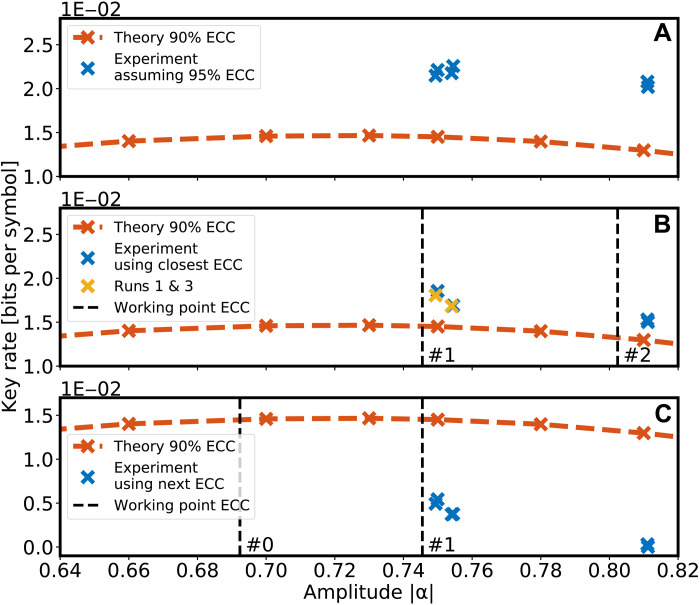
Composable finite-size key rates, using the experimental data and different error correction models. (**A**) We assume that, at any amplitude and resulting BER, an ECC with an efficiency of 95% is available. (**B**) We use the fixed leakage of the closest implemented LDPC code, i.e., ECC#1 for ∣α∣≈0.75 and ECC#2 for ∣α∣≈0.81. The highlighted measurement runs are further analyzed in Fig. 7. (**C**) We use the fixed leakage of the next, less optimal LDPC code, i.e., ECC#0 for ∣α∣≈0.75 and ECC#1 for ∣α∣≈0.81. To find the optimal amplitude for the system, we precalculated key rates for conservative system parameters: $N=1\times 10^{9}$, $r_{\text{test}}=0.25$, T=0.49, η=0.72, $\nu_{\text{el}}=0.14$, $\xi_{\text{A}}=0.3\%$, and β=0.9, denoted as theoretical key rates.

We operated the system at two different working points close to both the optimal amplitude maximizing the key rate and close to the implemented LDPC codes rendering efficient error correction and key generation feasible (see Fig. 6). For each of the six runs, the key rate was bounded with the security statement of Eq. 5 with the ϵ parameters set to $\epsilon_{\text{ET}}=\frac{1}{10}\times 10^{-10}$, $\epsilon_{\text{AT}}=\frac{7}{10}\times 10^{-10}$, $\bar{\epsilon }=\frac{7}{10}\times 10^{-10}$, $\epsilon_{\text{EC}}=\frac{2}{10}\times 10^{-10}$, and $\epsilon_{\text{PA}}=\frac{1}{10}\times 10^{-10}$, leading to a total security parameter of $\epsilon =\epsilon_{\text{EC}}+\text{max}\left\{\frac{1}{2}\epsilon_{\text{PA}}+\bar{\epsilon },\epsilon_{\text{ET}}+\epsilon_{\text{AT}}\right\}=1\times 10^{-10}$. Furthermore, the key rate was calculated with three different error correction models. At first and as commonly used in theoretical work and experiments without implemented error correction, the costs $\delta_{\text{leak}}$ were calculated by assuming a 95% efficient ECC with respect to the BSC capacity at the experimental BER. For the other two key rates, we do not assume the efficiency of a potential ECC. Instead, we subtracted the leakage of one of the implemented LDPC codes. For the second key rate, we used the LDPC code with its threshold BER closest to the measured BER (e.g., ECC#1 for ∣α∣≈0.75). For the third key rate, we used the next, less optimal implemented code (e.g., ECC#0 for the same amplitude).

The highest key rates were achieved when assuming a 95% ECC, which is also the common figure of merit to compare the performance to other QKD systems without implemented error correction. Doing so, we achieved composable finite-size key rates up to $2.26\times 10^{-2}$ bits per symbol against collective i.i.d. attacks. If one wants to characterize the performance of a QKD system that includes error correction and key generation, one has to study the key rates established with the true error correction costs. Because the implemented LDPC codes have a fixed code rate $R=1-\delta_{\text{leak}}$ with a fixed leakage $\delta_{\text{leak}}$ per bit, their efficiency $\beta =\frac{R}{C}$ reduces when increasing the amplitude and hence the channel capacity *C* of the system. Consequently, the closest LDPC codes operating with an efficiency of ~89% (see Table 1) achieve higher nominal key rates compared to the less optimal codes with a lower efficiency of ~78%. However, we have to operate the system close to the threshold BER of the LDPC codes to achieve high efficiencies. Being so close to its threshold, the error correction may fail to correct some of its blocks, leading to a nonzero FER. As discussed in the next chapter, sacrificing the nominal key rate for the benefit of reducing the FER can optimize the actual extractable secret key after error correction.

**Table 1.** Extracted secret keys and the influence of error correction for all six runs. The runs have similar experimental observables, leading to similar key rate estimates for each ECC model in Fig. 6. However, taking frame errors of error correction into account, the extractable key length is highly susceptible to the FER. Each run was either corrected with the ECC with a threshold BER closest to the experimental data or with the next, nonoptimal ECC with a higher threshold BER. The longest key of 1.6 MB was extracted for moderating between FER and efficiency β.

	Closest ECC			Next ECC		
∣α∣	FER [%]	β [%]	Key [MB]	FER [%]	β [%]	Key [MB]
0.7494	16.00	89.48	0.19	0.00	78.29	0.74
0.7499	14.37	89.42	0.52	0.00	78.24	0.81
0.7540	5.89	88.23	1.60	0.00	77.20	0.55
0.7545	6.18	88.77	1.56	0.00	77.67	0.56
0.8111	5.07	88.13	1.38	0.00	77.12	0.04
0.8112	9.79	88.29	0.49	0.01	77.25	0.00

### Error correction and key extraction

To study the impact of the implemented error correction and privacy amplification modules, we rewrite the key rate equation of the security statement in its leftover hashing lemma form, describing the extractable secret key length in bits

$$l\leq n\left[\underset{\rho \in S^{E\&A}}{\text{min}}H{\left(X|E\right)}_{\rho }-\delta \left(\bar{\epsilon }\right)-\Delta \left(\omega \right)\right]-{\text{leak}}_{\text{EC}}-2{\text{log}}_{2}\left(\frac{1}{\epsilon_{\text{PA}}}\right)$$ (6)

Here, the conditional von Neumann entropy H(X∣E) and both the correction terms for dimension reduction Δ(ω) and the asymptotic equipartition property $\delta \left(\bar{\epsilon }\right)$ are still retrieved solving the security proof’s SDP problem. The total error correction costs ${\text{leak}}_{\text{EC}}$ are now given by the number of disclosed bits in the LDPC module and the length of the confirmation hash t=128 bits. This leads to a correctness of the key of $\epsilon_{\text{cor}}=\epsilon_{\text{EC},\text{imp}}=\frac{m}{t}\cdot 2^{-t}<10^{-31}$, where *m* is the block length that is confirmed, i.e., the number of bits input to error correction (*69*). The secrecy of the key is guaranteed by subtracting $2{\text{log}}_{2}\left(\frac{1}{\epsilon_{\text{PA}}}\right)=100$ bits from the secret key length in the privacy amplification module, leading to $\epsilon_{\text{PA},\text{imp}}=2^{-50}$ and $\epsilon_{\text{sec}}=\text{max}\left\{\frac{1}{2}\epsilon_{\text{PA},\text{imp}}+\bar{\epsilon },\epsilon_{\text{ET}}+\epsilon_{\text{AT}}\right\}=\frac{4}{5}\times 10^{-10}$. For the whole implemented QKD protocol, including the QRNG with $\epsilon_{Q}=2^{-100}$, we hence achieve a total security parameter of $\epsilon_{\text{imp}}=\epsilon_{Q}+\epsilon_{\text{sec}}+\epsilon_{\text{cor}}<1\times 10^{-10}$. The results are listed in Table 1, where we achieve to generate composable secret keys with up to 1.6-MB length for a finite block size of $N=1.2\times 10^{9}$.

As stated in Introduction, it seems common in the literature to estimate the impact of frame errors by just adding a scaling factor (1−FER) to the key rate equation. This would allow for optimizing key rates by operating the system close to the threshold of the error correction, increasing its efficiency β on the cost of a high FER. However, one has to be careful on how frame errors are treated in the implemented QKD protocol and how that affects the different terms in the used security statement. In our case, we chose the conservative approach where, to have a secure key shared between Alice and Bob, the blocks that have failed error correction are fully disclosed between them. This does not reduce the key length in the leftover hashing lemma (Eq. 6) with a scaling factor but increases the number of disclosed bits ${\text{leak}}_{\text{EC}}$ that have to be subtracted from the secret key. In contrast to a pure scaling factor, this subtraction can quickly lead to zero key lengths when increasing the FER and hence disclosing too many blocks.

**Fig. 7.**
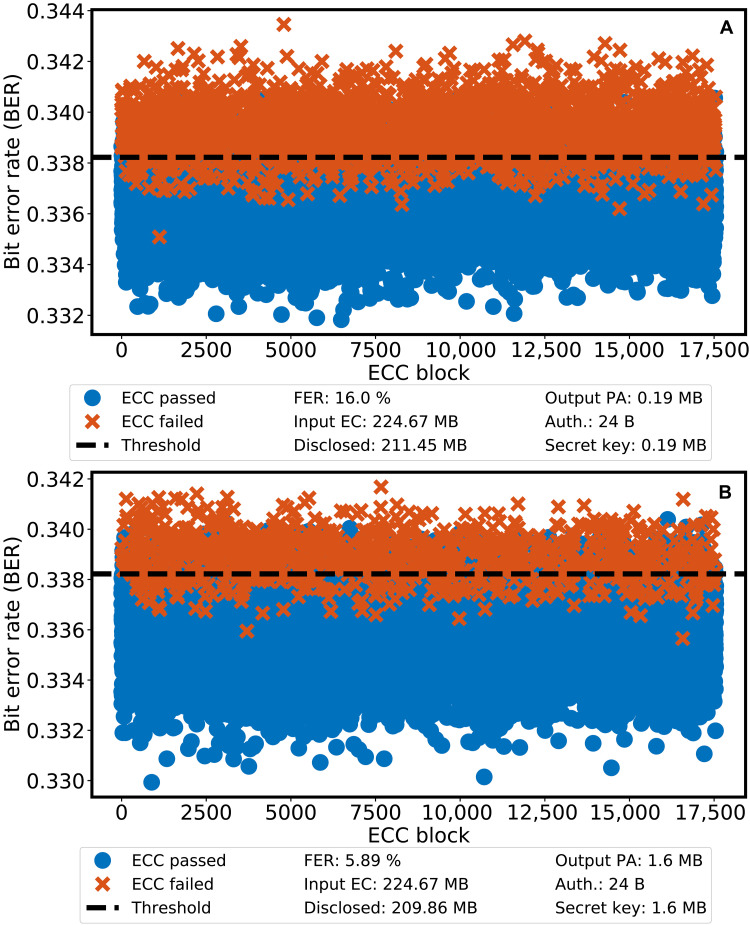
Detailed analysis of the postprocessing pipeline. The closest LDPC code ECC#1 is applied to (**A**) run 1 and (**B**) run 3. After 25% of the sent states are publicly disclosed, error correction (EC) is performed on the remaining $\approx 0.9\times 10^{9}$ private states with $\approx 1.8\times 10^{9}$ private bits. Each block that cannot be corrected adds its whole length of $L_{\text{in}}=102,400$ bits to the disclosed bits, reducing the extracted secret key after privacy amplification (PA). For each run, the AIT-QPS pipeline stores 2x96 bits for the next authentication (auth.) round.

As shown in Table 1 for the first two runs, operating the system with LDPC codes with a high efficiency and FER provides less key than using a (at first glance nonoptimal) LDPC code that only has an efficiency of 78% but can correct all blocks. For runs 3 and 4, we slightly increased the amplitude to moderate between a lower FER with sufficiently high efficiency, leading to the highest extracted key lengths. We also operated the system at a different working point (runs 5 and 6), where we also achieved high key lengths for moderate FERs. Please note the higher FER for the last run reducing the key length. This is due to a small drift in the modulators preparing the states, leading to a similar mean amplitude, but a deviation in the X/P bit streams after applying the key map projection. Like that, the P bit stream has a BER closer to the threshold BER, leading to more frame errors in correcting P (see table S1 in the Supplementary Materials). The effect of the frame errors is also illustrated in Fig. 7.

## DISCUSSION

We present a CV QKD system using polarization encoding and discrete QPSK modulation designed for future applications in urban atmospheric channels. In a proof-of-principle experiment in the laboratory, we implemented the protocol of a recently developed security proof that allows for composable finite-size key rates against i.i.d. collective attacks for discrete modulation without requiring any Gaussian assumptions. We achieved composable finite-size key rates upon acceptance with up to $2.26\times 10^{-2}$ bits per symbol. With the implemented QKD pipeline, including the QRNG, error correction and privacy amplification, we were able to generate secret keys up to 1.6 MB for $N=1.2\times 10^{9}$ sent states with a total security parameter of $\epsilon_{\text{imp}}<1\times 10^{-10}$. We call the keys composable upon acceptance to highlight the fact that we used the honest implementation in the laboratory to both define the nonunique acceptance sets and run the QKD protocol with the statistics of the same measurement run. For a next practical implementation over an untrusted channel, we foresee testing the measured statistics against beforehand defined acceptance sets.

Having a transmitted LO is an active design choice for future atmospheric links as the LO and the signal share the same spatial mode and will experience the same wavefront distortions. They are then autocompensated when optically interfering during Stokes detection (*14*). Using a transmitted LO also allows for a low noise operation without the typical challenge of residual phase noise in systems with a true LO (*10*). However, to prevent hacking attacks on the transmitted LO (*70*, *71*), we foresee upgrading the power measurement to a Stokes ${\hat{S}}_{3}$ measurement as described in (*48*). Furthermore, we plan to add a second laser at Bob and switch to local vacuum measurements at random times, verifying the transmitted vacuum reference.

In the current system, the communication of the AIT-QPS pipeline is already authenticated using polynomial universal hashing with a preshared key (*72*). To authenticate the whole classical communication of the protocol, one has to use parts of the generated key for the authentication in the next run *n* (*73*). The new security parameter is then given by $\epsilon <n\left(\epsilon_{\text{imp}}+\epsilon_{\text{auth}}\right)$ with $\epsilon_{\text{auth}}=\frac{c}{a}\cdot 2^{-a}$ and a hash size of a=96 bits, linearly increasing with each run (*69*). The length of the total classical communication (in bits) to be authenticated is denoted with *c*. With c≈450 MB in the AIT-QPS pipeline and c≈30 MB during the optical quantum state exchange phase, including the latter in the authenticated communication will merely affect $\epsilon_{\text{auth}}<10^{-21}$.

We also studied the effect of frame errors on the extracted key length not only when using a scaling factor in front of the key rate but also when sticking to the security statement of the protocol. In the context of asymptotic key rates, our approach is similar to include the frame errors with $K=\left(1-\text{FER}\right)\beta I_{\text{AB}}-\chi_{\text{BE}}$, being seen as a lower bound when properly taking them into account (*25*). This treatment is pessimistic as disclosed blocks currently do not add 0 bits to the key length but actually add a negative contribution due to the fixed conditional entropy. Another treatment could be to not disclose frame errors but to discard them completely. To do this properly, one has to include the postselection of frame errors in the postprocessing map of the protocol [see (*28*)], similar to the already included phase space postselection. Discarded blocks would then not appear in the error correction costs while being included in a modified smooth min entropy. A similar modification was done in the framework of composable security for Gaussian-modulated protocols (*11*, *44*). Furthermore, using rate-adaptive codes with a higher efficiency and lower FER will increase the performance of the system (*74*, *75*). We also plan to study the effect of postselection as a reduced data stream in the postprocessing pipeline will relax hardware requirements.

## MATERIALS AND METHODS

### Temporal mode

As more and more advanced techniques from classical DSP are applied to CV QKD systems to enhance their performance, the consequences of such algorithms on the underlying quantum optical definitions have to be taken into account. In particular, filter operations acting on the spectral properties of the signal are also changing the temporal mode and with that the definitions of the sent and measured quantum states. As outlined in the following, this could lead to a mode mismatch between the sender and the receiver, which results in hidden additional detection losses, rendering estimated key rates insecure.

**Fig. 8.**
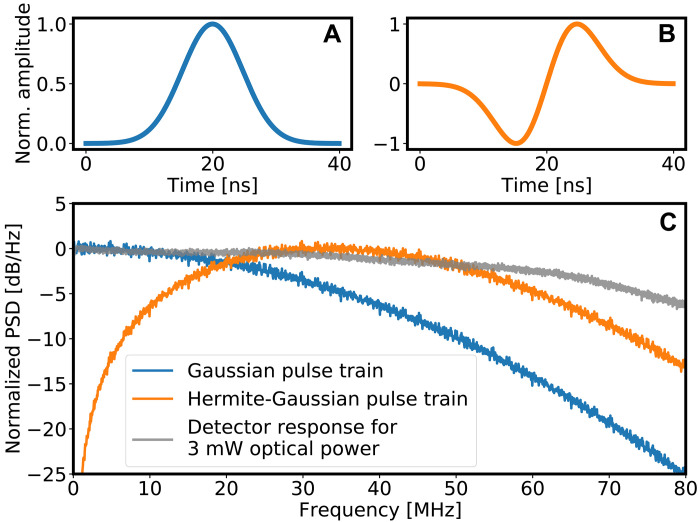
Temporal modes in the time and spectral domain. (**A**) Gaussian mode. (**B**) First-order Hermite-Gaussian mode. (**C**) rf spectra of a QPSK signal trace encoded in either Gaussian or Hermite-Gaussian pulses, showing the low-frequency suppression of Hermite-Gaussian modes. The spectral response of the used homodyne detectors is flat and has a higher bandwidth than the QKD signal trace, allowing for proper mode conversion from the optical to the electrical domain. For the detector response, the electronic noise spectrum is subtracted.

In this work, the quantum states are temporally encoded in modified Hermite polynomials of first order [Hermite-Gaussian pulses; see (*76*)] defined by

$$\Gamma \left(t\right)=\frac{\sqrt{e}\cdot t}{\sigma }\cdot \text{exp}\left[-\frac{1}{2}{\left(\frac{t}{\sigma }\right)}^{2}\right]$$ (7)

with σ≈5 ns so that there is no interference between the different pulse windows. Similar to systems using frequency upshifted RRC filtering (*77*), the rf spectra of such an encoded signal is moved away from the low-frequency regime (see Fig. 8). The additional loss introduced by omitting the residual energy of the Hermite-Gaussian modes in the passband of the high-pass filters below 130 kHz is in the order of $10^{-8}$ and currently neglected. Furthermore, the pulse spectrum acts as an additional intrinsic filter against low-frequency noise, reducing the preparation noise of the system by one order of magnitude with respect to a standard Gaussian pulse.

By defining the pulse shape, we also define the temporal mode Γ(t) of the quantum states with the corresponding broadband-mode operators ${\hat{A}}_{\Gamma }^{†}=\int dt\;\Gamma \left(t\right){\hat{a}}^{†}\left(t\right)$ (*78*). They also fulfill the bosonic commutation relation $\left[{\hat{A}}_{\Gamma }^{†},{\hat{A}}_{\Gamma }\right]=1$, allowing us to use the same framework as known from the standard single-mode treatment by replacing the single-mode ladder operators $\left\{{\hat{a}}_{\omega }^{†},{\hat{a}}_{\omega }\right\}$ with their broadband counterpart $\left\{{\hat{A}}_{\Gamma }^{†},{\hat{A}}_{\Gamma }\right\}$. However, one has to ensure that the measured quadrature operator ${\hat{X}}_{\Lambda }$ and its temporal mode Λ(t) are defined according to the sent temporal mode; otherwise, the mode mismatch between both modes leads to a decreased interferometric visibility and increased receiver losses.

For time-resolved homodyne detection, the measured mode is dependent on the temporal mode of the LO, the ADC oversampling, the bandwidth of the detectors, and the used linear DSP algorithm. We are using a continuous-wave LO and dense ADC oversampling with $f_{\text{ADC}}=1.25$ GHz. By taking the weighted average over the oversampled pulse window and ensuring that the detector bandwidth is larger than the signal bandwidth, we can ensure a high mode-matching degree between both temporal modes (*79*).

## Supplementary Materials

### This PDF file includes:

Supplementary Text Table S1 References
