# Supplementary material for: Composable free-space continuous-variable quantum key distribution using discrete modulation
Source: Sci Adv. 2026 Jun 12;12(24):eadv1440. doi: 10.1126/sciadv.adv1440 (PMC13262621; doi:10.1126/sciadv.adv1440)
Supplement: Supplementary file 1 — Supplementary Text Table S1 References [file sciadv.adv1440_sm.pdf]

Science Advances
AAAS

Supplementary Materials for

Composable free-space continuous-variable quantum key distribution using discrete modulation

Kevin Jaksch *et al.*

Corresponding author: Kevin Jaksch, kevin.jaksch@fau.de;
Christoph Marquardt, christoph.marquardt@fau.de

Sci. Adv. **12**, eadv1440 (2026)
DOI: 10.1126/sciadv.adv1440

This PDF file includes:

Supplementary Text
Table S1
References

Supplementary Text: Optimization problem for the security proof

In this section, we give additional information about the chosen observables, the acceptance set, and the semi-definite program we solve to obtain secure key rates. We start by introducing the observables used. While Alice's and Bob's heterodyne measurement allows them to measure the x and p quadrature of the incoming signals, for reasons of security analysis, it turns out that it is advantageous to use displaced versions of the photon number $\hat{n}$ and the squared photon number $\hat{n}^2$ as observables. We denote them by $\hat{n}_{\beta_j} := \hat{D}(\beta_j)\hat{n}\hat{D}^\dagger(\beta_j)$ and $\hat{n}^2_{\beta_j} := \hat{D}(\beta_j)\hat{n}^2\hat{D}^\dagger(\beta_j)$ for $j \in \{0, 1, 2, 3\}$ and $\beta_j := \sqrt{T\eta}\alpha_j$. $\hat{D}(\gamma)$ is the displacement operator. Note that this does not mean we need to know the systems' η or α_j exactly and beforehand. We simply use the expectation according to our model and apply the displacement to obtain our displaced observables. In case the actual (unknown) values differ, we will see this in our observations. Those observables are directly related to the heterodyne measurement outcomes [see, for example, Ref. (*80*)].

Then, following the theory in Ref. (*31, 62*), we arrive at the following optimization problem in Eq. S1. From the acceptance testing theorem [Theorem 4 in (*31*)], we obtain $\mu_X := \sqrt{\frac{||X||^2_\infty}{2m_X} \ln\left(\frac{2}{\epsilon_{\mathrm{AT}}}\right)}$ for $X \in \{\hat{n}_{\beta_j}, \hat{n}^2_{\beta_j}\}$ and we obtain as a function of the bounded detection range M [see also Ref. (*31*)] the following $||\hat{n}_{\beta_j}||_\infty = M^2 - \frac{1}{2}$, and $||\hat{n}^2_{\beta_j}||_\infty = M^4 - \frac{1}{2}M^2$. Using non-unique acceptance testing, we allow the measurement to deviate from the expected statistics $\langle X \rangle$ by $t_X = t\mu_X$. Furthermore, w is the weight introduced for energy testing, ρ_{A} is the reduced density matrix on Alice's system and N, P are optimization variables originating from the trace-norm constraint on the marginal state ρ_{A} [see (*31*)]. The optimization is over the density matrix on the chosen finite dimensional Hilbert space, denoted by $\bar{\rho}$.

The measured parameters for all six runs and four QPSK states, as used for the numerical SDP problem, are depicted in table S1.

$$
\begin{aligned}
&\beta := \min \ f(\bar{\rho}) \\
&\text{s.t.} \\
&\quad \mathrm{Tr}\,[P] + \mathrm{Tr}\,[N] \leq 2\sqrt{w} \\
&\quad P \geq \mathrm{Tr}_B\,[\overline{\rho}] - \rho_A \\
&\quad N \geq -\left(\mathrm{Tr}_B\,[\bar{\rho}] - \rho_A\right) \\
&\quad \mathrm{Tr}\left[\left(-\frac{1}{p_j}|j\rangle\langle j| \otimes \hat{n}_{\beta_j}\right)\bar{\rho}\right] \leq \left(\mu_{\hat{n}_{\beta_j}} + t_{\hat{n}_{\beta_j}}\right) - \langle \hat{n}_{\beta_j}\rangle + w||\hat{n}_{\beta_j}||_\infty \\
&\quad \mathrm{Tr}\left[\left(\frac{1}{p_j}|j\rangle\langle j| \otimes \hat{n}_{\beta_j}\right)\bar{\rho}\right] \leq \left(\mu_{\hat{n}_{\beta_j}} + t_{\hat{n}_{\beta_j}}\right) + \langle \hat{n}_{\beta_j}\rangle \\
&\quad \mathrm{Tr}\left[\left(-\frac{1}{p_j}|j\rangle\langle j| \otimes \hat{n}^2_{\beta_j}\right)\bar{\rho}\right] \leq \left(\mu_{\hat{n}^2_{\beta_j}} + t_{\hat{n}^2_{\beta_j}}\right) - \langle \hat{n}^2_{\beta_j}\rangle + w||\hat{n}^2_{\beta_j}||_\infty \\
&\quad \mathrm{Tr}\left[\left(\frac{1}{p_j}|j\rangle\langle j| \otimes \hat{n}^2_{\beta_j}\right)\bar{\rho}\right] \leq \left(\mu_{\hat{n}^2_{\beta_j}} + t_{\hat{n}^2_{\beta_j}}\right) + \langle \hat{n}^2_{\beta_j}\rangle \\
&\quad 1 - w \leq \mathrm{Tr}\,[\overline{\rho}] \leq 1 \\
&\quad \bar{\rho}, P, N \geq 0
\end{aligned}
\tag{S1}
$$

Table S1: **Measured parameters for all six runs and four QPSK states as used for the numerical SDP problem in (*31*)**. n: Total number of private states. N_{tot}: Total number of sent states. T: Channel transmission. η: Receiver efficiency. ν_{el}: Detector noise. I_T: Relative outliers for energy test. BER X/P: Measured BER after applying key map. α_j: Sending amplitude for each QPSK state. $\langle \hat{n}_{\beta_j} \rangle$: Mean displaced photon number for each QPSK state. $\langle \hat{n}^2_{\beta_j} \rangle$: Mean squared displaced photon number for each QPSK state.

Run	n [10^8]	N_{tot} [10^9]	T [1]	η [1]	ν_{el} [1]	I_T [10^{-8}]	BER X [1]	BER P [1]
1	8.9866	1.1982	0.4950	0.72	0.1350	0.6677	0.3378	0.3368
2	8.9866	1.1982	0.4950	0.72	0.1351	0.0000	0.3376	0.3368
3	8.9866	1.1982	0.4959	0.72	0.1354	0.6677	0.3367	0.3357
4	8.9866	1.1983	0.4938	0.72	0.1348	0.3338	0.3367	0.3362
5	8.9866	1.1982	0.4943	0.72	0.1349	0.0000	0.3253	0.3245
6	8.9866	1.1982	0.4914	0.72	0.1354	0.0000	0.3247	0.3261

Run	α_0 [1]	α_1 [1]	α_2 [1]	α_3 [1]
1	0.5266+0.5210i	0.5303-0.5418i	-0.5299+0.5420i	-0.5261-0.5211i
2	0.5263+0.5221i	0.5312-0.5414i	-0.5314+0.5420i	-0.5263-0.5215i
3	0.5289+0.5255i	0.5338-0.5442i	-0.5343+0.5444i	-0.5286-0.5257i
4	0.5319+0.5282i	0.5335-0.5404i	-0.5336+0.5406i	-0.5317-0.5280i
5	0.5687+0.5628i	0.5758-0.5871i	-0.5752+0.5868i	-0.5685-0.5630i
6	0.5657+0.5678i	0.5862-0.5745i	-0.5860+0.5745i	-0.5660-0.5681i

Run /	$\langle \hat{n}_{\beta_j} \rangle$ [10^{-3}]				$\langle \hat{n}^2_{\beta_j} \rangle$ [10^{-3}]			
Symbol j	0	1	2	3	0	1	2	3
1	0.7486	0.4292	0.0144	1.5718	7.0560	7.3561	6.4495	5.9361
2	0.9354	0.1293	0.0390	0.9096	6.1404	7.0781	7.1981	5.9385
3	0.9458	0.4128	0.0691	1.1511	5.2876	6.9297	6.3640	6.8303
4	1.0900	0.1365	0.0197	1.1424	6.2311	8.1145	6.1596	6.6191
5	0.8604	0.2875	0.3552	1.8903	5.9607	7.2291	8.2480	6.1189
6	1.0567	0.2228	0.2246	1.4379	7.4074	7.3594	7.2678	7.2334

REFERENCES

1. T. C. Ralph, Continuous variable quantum cryptography. *Phys. Rev. A* **61**, 010303 (1999).

2. F. Grosshans, P. Grangier, Continuous variable quantum cryptography using coherent states. *Phys. Rev. Lett.* **88**, 057902 (2002).

3. C. Silberhorn, T. C. Ralph, N. Lütkenhaus, G. Leuchs, Continuous variable quantum cryptography: Beating the 3 dB loss limit. *Phys. Rev. Lett.* **89**, 167901 (2002).

4. I. Khan, B. Stiller, K. Jaksch, N. Jain, C. Peuntinger, K. Günthner, T. Röthlingshöfer, D. Elser, C. Marquardt, G. Leuchs, Towards continuous-variable quantum key distribution at GHz rates, poster presented at the 5th Annual Conference on Quantum Cryptography (QCrypt), Tokyo, Japan, 28 September to 2 October 2015.

5. S. Pirandola, U. L. Andersen, L. Banchi, M. Berta, D. Bunandar, R. Colbeck, D. Englund, T. Gehring, C. Lupo, C. Ottaviani, J. L. Pereira, M. Razavi, J. S. Shaari, M. Tomamichel, V. C. Usenko, G. Vallone, P. Villoresi, P. Wallden, Advances in quantum cryptography. *Adv. Opt. Photonics* **12**, 1012–1236 (2020).

6. Y. Zhang, Y. Bian, Z. Li, S. Yu, H. Guo, Continuous-variable quantum key distribution system: Past, present, and future. *Appl. Phys. Rev.* **11**, 011318 (2024).

7. F. Laudenbach, C. Pacher, C.-H. F. Fung, A. Poppe, M. Peev, B. Schrenk, M. Hentschel, P. Walther, H. Hübel, Continuous-variable quantum key distribution with Gaussian modulation—The theory of practical implementations. *Adv. Quantum Technol.* **1**, 1800011 (2018).

8. P. Jouguet, S. Kunz-Jacques, A. Leverrier, P. Grangier, E. Diamanti, Experimental demonstration of long-distance continuous-variable quantum key distribution. *Nat. Photonics* **7**, 378–381 (2013).

9. Y. Zhang, Z. Chen, S. Pirandola, X. Wang, C. Zhou, B. Chu, Y. Zhao, B. Xu, S. Yu, H. Guo, Long-distance continuous-variable quantum key distribution over 202.81 km of fiber. *Phys. Rev. Lett.* **125**, 010502 (2020).

10. A. A. E. Hajomer, I. Derkach, N. Jain, H.-M. Chin, U. L. Andersen, T. Gehring, Long-distance continuous-variable quantum key distribution over 100-km fiber with local local oscillator. *Sci. Adv.* **10**, eadi9474 (2024).

11. N. Jain, H.-M. Chin, H. Mani, C. Lupo, D. S. Nikolic, A. Kordts, S. Pirandola, T. B. Pedersen, M. Kolb, B. Ömer, C. Pacher, T. Gehring, U. L. Andersen, Practical continuous-variable quantum key distribution with composable security. *Nat. Commun.* **13**, 4740 (2022).

12. A. A. E. Hajomer, N. Jain, H. Mani, H.-M. Chin, U. L. Andersen, T. Gehring, Modulation leakage-free continuous-variable quantum key distribution. *npj Quantum Inf.* **8**, 136 (2022).

13. C. Wittmann, J. Fürst, C. Wiechers, D. Elser, H. Häseler, N. Lütkenhaus, G. Leuchs, Witnessing effective entanglement over a 2 km fiber channel. *Opt. Express* **18**, 4499–4509 (2010).

14. B. Heim, C. Peuntinger, N. Killoran, I. Khan, C. Wittmann, C. Marquardt, G. Leuchs, Atmospheric continuous-variable quantum communication. *New J. Phys.* **16**, 113018 (2014).

15. S. Kleis, M. Rueckmann, C. G. Schaeffer, Continuous variable quantum key distribution with a real local oscillator using simultaneous pilot signals. *Opt. Lett.* **42**, 1588–1591 (2017).

16. S. Ghorai, P. Grangier, E. Diamanti, A. Leverrier, Asymptotic security of continuous-variable quantum key distribution with a discrete modulation. *Phys. Rev. X* **9**, 021059 (2019).

17. A. Denys, P. Brown, A. Leverrier, Explicit asymptotic secret key rate of continuous-variable quantum key distribution with an arbitrary modulation. *Quantum* **5**, 540 (2021).

18. E. Kaur, S. Guha, M. M. Wilde, Asymptotic security of discrete-modulation protocols for continuous-variable quantum key distribution. *Phys. Rev. A* **103**, 012412 (2021).

19. F. Roumestan, A. Ghazisaeidi, J. Renaudier, L. T. Vidarte, A. Leverrier, E. Diamanti, P. Grangier, Shaped constellation continuous variable quantum key distribution: Concepts, methods and experimental validation. *J. Lightwave Technol.* **42**, 5182–5189 (2024).

20. Y. Pan, H. Wang, Y. Shao, Y. Pi, Y. Li, B. Liu, W. Huang, B. Xu, Experimental demonstration of high-rate discrete-modulated continuous-variable quantum key distribution system. *Opt. Lett.* **47**, 3307–3310 (2022).

21. A. A. E. Hajomer, C. Bruynsteen, I. Derkach, N. Jain, A. Bomhals, S. Bastiaens, U. L. Andersen, X. Yin, T. Gehring, Continuous-variable quantum key distribution at 10 GBaud using an integrated photonic-electronic receiver. *Optica* **11**, 1197–1204 (2024).

22. H. Wang, Y. Li, Y. Pi, Y. Pan, Y. Shao, L. Ma, Y. Zhang, J. Yang, T. Zhang, W. Huang, B. Xu, Sub-Gbps key rate four-state continuous-variable quantum key distribution within metropolitan area. *Commun. Phys.* **5**, 162 (2022).

23. Y. Tian, Y. Zhang, S. Liu, P. Wang, Z. Lu, X. Wang, Y. Li, High-performance long-distance discrete-modulation continuous-variable quantum key distribution. *Opt. Lett.* **48**, 2953–2956 (2023).

24. Y. Xu, T. Wang, L. Li, H. Zhao, P. Huang, G. Zeng, Simultaneous continuous-variable quantum key distribution and classical optical communication over a shared infrastructure. *Appl. Phys. Lett.* **123**, 154001 (2023).

25. S. J. Johnson, A. M. Lance, L. Ong, M. Shirvanimoghaddam, T. C. Ralph, T. Symul, On the problem of non-zero word error rates for fixed-rate error correction codes in continuous variable quantum key distribution. *New J. Phys.* **19**, 023003 (2017).

26. P. J. Coles, E. M. Metodiev, N. Lütkenhaus, Numerical approach for unstructured quantum key distribution. *Nat. Commun.* **7**, 11712 (2016).

27. A. Winick, N. Lütkenhaus, P. J. Coles, Reliable numerical key rates for quantum key distribution. *Quantum* **2**, 77 (2018).

28. J. Lin, T. Upadhyaya, N. Lütkenhaus, Asymptotic security analysis of discrete-modulated continuous-variable quantum key distribution. *Phys. Rev. X* **9**, 041064 (2019).

29. J. Lin, N. Lütkenhaus, Trusted detector noise analysis for discrete modulation schemes of continuous-variable quantum key distribution. *Phys. Rev. Appl.* **14**, 064030 (2020).

30. T. Upadhyaya, T. van Himbeeck, J. Lin, N. Lütkenhaus, Dimension reduction in quantum key distribution for continuous- and discrete-variable protocols. *PRX Quantum* **2**, 020325 (2021).

31. F. Kanitschar, I. George, J. Lin, T. Upadhyaya, N. Lütkenhaus, Finite-size security for discrete-modulated continuous-variable quantum key distribution protocols. *PRX Quantum* **4**, 040306 (2023).

32. C. Lupo, Y. Ouyang, Quantum key distribution with nonideal heterodyne detection: Composable security of discrete-modulation continuous-variable protocols. *PRX Quantum* **3**, 010341 (2022).

33. S. Bäuml, C. Pascual-García, V. Wright, O. Fawzi, A. Acín, Security of discrete-modulated continuous-variable quantum key distribution. *Quantum* **8**, 1418 (2024).

34. C. Pascual-García, S. Bäuml, M. Araújo, R. Liss, A. Araújo, Improved finite-size key rates for discrete-modulated continuous variable quantum key distribution under coherent attacks. arXiv:2407.03087 [quant-ph] (2024).

35. I. W. Primaatmaja, W. Y. Kon, C. Lim, Discrete-modulated continuous-variable quantum key distribution secure against general attacks. arXiv: 2409.02630 [quant-ph] (2024).

36. F. Kanitschar, C. Pacher, Security of multi-user quantum key distribution with discrete-modulated continuous-variables. arXiv:2406.14610 [quant-ph] (2024).

37. K. Günthner, I. Khan, D. Elser, B. Stiller, Ö. Bayraktar, C. R. Müller, K. Saucke, D. Tröndle, F. Heine, S. Seel, P. Greulich, H. Zech, B. Gütlich, S. Philipp-May, C. Marquardt, G. Leuchs, Quantum-limited measurements of optical signals from a geostationary satellite. *Optica* **4**, 611–616 (2017).

38. D. Dequal, L. Trigo Vidarte, V. Roman Rodriguez, G. Vallone, P. Villoresi, A. Leverrier, E. Diamanti, Feasibility of satellite-to-ground continuous-variable quantum key distribution. *npj Quantum Inf.* **7**, 3 (2021).

39. M. T. Sayat, B. Shajilal, S. P. Kish, S. M. Assad, T. Symul, P. K. Lam, N. J. Rattenbury, J. E. Cater, Satellite-to-ground continuous variable quantum key distribution: The Gaussian and discrete modulated protocols in low Earth orbit. *IEEE Trans. Commun.* **72**, 3244–3255 (2024).

40. S.-Y. Shen, M.-W. Dai, X.-T. Zheng, Q.-Y. Sun, G.-C. Guo, Z.-F. Han, Free-space continuous-variable quantum key distribution of unidimensional Gaussian modulation using polarized coherent states in an urban environment. *Phys. Rev. A* **100**, 012325 (2019).

41. S.-Y. Shen, X.-T. Zheng, G.-C. Guo, Z.-F. Han, Atmospheric effect study of continuous-variable quantum key distribution. *Opt. Lett.* **45**, 2592–2595 (2020).

42. V. C. Usenko, B. Heim, C. Peuntinger, C. Wittmann, C. Marquardt, G. Leuchs, R. Filip, Entanglement of Gaussian states and the applicability to quantum key distribution over fading channels. *New J. Phys.* **14**, 093048 (2012).

43. L. Ruppert, C. Peuntinger, B. Heim, K. Günthner, V. C. Usenko, D. Elser, G. Leuchs, R. Filip, C. Marquardt, Fading channel estimation for free-space continuous-variable secure quantum communication. *New J. Phys.* **21**, 123036 (2019).

44. S. Pirandola, Limits and security of free-space quantum communications. *Phys. Rev. Res.* **3**, 013279 (2021).

45. K. Gümüş, J. dos Reis Frazão, V. van Vliet, S. van der Heide, M. van den Hout, A. Albores-Mejia, T. Bradley, C. Okonkwo, "Adaptive reconciliation for experimental continuous-variable quantum key distribution over a turbulent free-space optical channel," in *Optical Fiber Communication Conference (OFC) 2024* (Optica Publishing Group, 2024), p. Th1C.4.

46. M. Sayat, M. Birch, O. Thearle, M. Copeland, E. Jager, F. Bennet, P. K. Lam, N. Rattenbury, J. Cater, "Experimental effects of turbulence on coherent states in a free-space channel using adaptive optics for continuous variable quantum key distribution," in *Quantum Computing, Communication, and Simulation IV*, P. R. Hemmer, A. L. Migdall, Eds. (International Society for Optics and Photonics, SPIE, 2024), vol. 12911, p. 1291107.

47. C. Peuntinger, B. Heim, C. R. Müller, C. Gabriel, C. Marquardt, G. Leuchs, Distribution of squeezed states through an atmospheric channel. *Phys. Rev. Lett.* **113**, 060502 (2014).

48. H. Häseler, T. Moroder, N. Lütkenhaus, Testing quantum devices: Practical entanglement verification in bipartite optical systems. *Phys. Rev. A* **77**, 032303 (2008).

49. A. A. E. Hajomer, F. Kanitschar, N. Jain, M. Hentschel, R. Zhang, N. Lütkenhaus, U. L. Andersen, C. Pacher, T. Gehring, Experimental composable key distribution using discrete-modulated continuous variable quantum cryptography. *Light Sci. Appl.* **14**, 255 (2025).

50. S. Q. Ng, F. Kanitschar, G. Zhang, C. Wang, Gigabit-rate quantum key distribution on integrated photonic chips. arXiv:2504.08298v1 [quant-ph] (2025).

51. M. Wu, Y. Pan, J. Li, H. Wang, L. Fan, Y. Shao, Y. Li, W. Huang, S. Yu, B. Xu, Y. Zhang, High-rate discrete-modulated continuous-variable quantum key distribution with composable security. arXiv:2503.11431v1 [quant-ph] (2025).

52. N. Korolkova, G. Leuchs, R. Loudon, T. C. Ralph, C. Silberhorn, Polarization squeezing and continuous-variable polarization entanglement. *Phys. Rev. A* **65**, 052306 (2002).

53. V. Josse, A. Dantan, A. Bramati, E. Giacobino, Entanglement and squeezing in a two-mode system: Theory and experiment. *J. Opt. B Quantum Semiclassical Opt.* **6**, S532–S543 (2004).

54. U. Leonhardt, H. Paul, Measuring the quantum state of light. *Prog. Quantum Electron.* **19**, 89–130 (1995).

55. M. Goy, J. Krause, Ö. Bayraktar, P. Ancsin, F. David, T. Dirmeier, N. Doell, J. Dwan, F. Fohlmeister, R. Freund, T. A. Goebel, J. Hilt, K. Jaksch, O. Kohout, T. Kopf, A. Krzic, M. Leipe, G. Leuchs, C. Marquardt, K. L. Mendez, A. Milde, S. Mishra, F. Moll, K. Paciorek, N. Pavlovic, S. Richter, M. Rothe, R. Rüddenklau, G. Sauer, M. Schell, J. Schreck, A. Schreier, S. Sharma, S. Spier, C. Spiess, F. Steinlechner, A. Tünnermann, H. Vural, N. Walenta, S. Weide, Ad-hoc hybrid-heterogeneous metropolitan-range quantum key distribution network. *New J. Phys.* **27**, 114510 (2025).

56. X.-T. Zheng, Q.-F. Zhang, J. Ling, G.-C. Guo, Z.-F. Han, Free-space continuous-variable quantum key distribution under high background noise. *npj Quantum Inf.* **11**, 52 (2025).

57. C. Gabriel, C. Wittmann, D. Sych, R. Dong, W. Mauerer, U. L. Andersen, C. Marquardt, G. Leuchs, A generator for unique quantum random numbers based on vacuum states. *Nat. Photonics* **4**, 711–715 (2010).

58. J. Y. Haw, S. M. Assad, A. M. Lance, N. H. Y. Ng, V. Sharma, P. K. Lam, T. Symul, Maximization of extractable randomness in a quantum random-number generator. *Phys. Rev. Appl.* **3**, 054004 (2015).

59. J. Carter, M. N. Wegman, Universal classes of hash functions. *J. Comput. Syst. Sci.* **18**, 143–154 (1979).

60. Y. Mansour, N. Nisan, P. Tiwari, "The computational complexity of universal hashing," in *Proceedings of the Twenty-Second Annual ACM Symposium on Theory of Computing, STOC '90* (Association for Computing Machinery, 1990), pp. 235–243.

61. J.-M. Conan, G. Rousset, P.-Y. Madec, Wave-front temporal spectra in high-resolution imaging through turbulence. *J. Opt. Soc. Am. A* **12**, 1559–1570 (1995).

62. F. P. Kanitschar, "Finite-size security proof for discrete-modulated CV-QKD protocols," thesis, TU Wien, Wien, Austria (2022).

63. F. Kanitschar, C. Pacher, Optimizing continuous-variable quantum key distribution with phase-shift keying modulation and postselection. *Phys. Rev. Appl.* **18**, 034073 (2022).

64. M. Frank, P. Wolfe, An algorithm for quadratic programming. *Nav. Res. Logist. Q.* **3**, 95–110 (1956).

65. M. Grant, S. Boyd, CVX: Matlab Software for Disciplined Convex Programming, version 2.1 (2014); http://cvxr.com/cvx/.

66. M. Grant, S. Boyd, "Graph implementations for nonsmooth convex programs" in *Recent Advances in Learning and Control, Lecture Notes in Control and Information Sciences*, V. Blondel, S. Boyd, H. Kimura, Eds. (Springer-Verlag Limited, 2008), pp. 95–110.

67. MOSEK ApS, The MOSEK optimization toolbox for MATLAB manual, version 9.0 (2019); http://docs.mosek.com/9.0/toolbox/index.html.

68. D. Tupkary, E. Y.-Z. Tan, N. Lütkenhaus, Security proof for variable-length quantum key distribution. *Phys. Rev. Res.* **6**, 023002 (2024).

69. A. Kržič, S. Sharma, C. Spiess, U. Chandrashekara, S. Töpfer, G. Sauer, L. J. González-Martín del Campo, T. Kopf, S. Petscharnig, T. Grafenauer, R. Lieger, B. Ömer, C. Pacher, R. Berlich, T. Peschel, C. Damm, S. Risse, M. Goy, D. Rieländer, A. Tünnermann, F. Steinlechner, Towards metropolitan free-space quantum networks. *npj Quantum Inf.* **9**, 95 (2023).

70. P. Jouguet, S. Kunz-Jacques, E. Diamanti, Preventing calibration attacks on the local oscillator in continuous-variable quantum key distribution. *Phys. Rev. A* **87**, 062313 (2013).

71. L. Fan, Y. Bian, M. Wu, Y. Zhang, S. Yu, Quantum hacking against discrete-modulated continuous-variable quantum key distribution using modified local oscillator intensity attack with random fluctuations. *Phys. Rev. Appl.* **20**, 024073 (2023).

72. C. Pacher, G. Lechner, C. Portmann, O. Maurhart, M. Peev, Efficient QKD postprocessing algorithms, poster presented at the *2nd Annual Conference on Quantum Cryptography (QCrypt)*, Singapore, Singapore, 10 to 14 September 2012.

73. J. Müller-Quade, R. Renner, Composability in quantum cryptography. *New J. Phys.* **11**, 085006 (2009).

74. C. Zhou, X. Wang, Z. Zhang, S. Yu, Z. Chen, H. Guo, Rate compatible reconciliation for continuous-variable quantum key distribution using Raptor-like LDPC codes. *Sci. China Phys. Mech. Atron.* **64**, 260311 (2021).

75. E. E. Cil, L. Schmalen, “Rate-adaptive protograph-based Raptor-like LDPC code for continuous-variable quantum key distribution,” in *Advanced Photonics Congress 2024* (Optica Publishing Group, 2024), p. JTu1A.51.

76. L. Michael, M. Ghavami, R. Kohno, “Multiple pulse generator for ultra-wideband communication using Hermite polynomial based orthogonal pulses,” in *2002 IEEE Conference on Ultra Wideband Systems and Technologies (IEEE Cat. No.02EX580)* (IEEE, 2002), pp. 47–51.

77. H.-M. Chin, N. Jain, D. Zibar, T. Gehring, U. L. Andersen, Effect of filter shape on excess noise performance in continuous variable quantum key distribution with Gaussian modulation. arXiv:1808.04573 [eess.SP] (2018).

78. B. Brecht, D. V. Reddy, C. Silberhorn, M. G. Raymer, Photon temporal modes: A complete framework for quantum information science. *Phys. Rev. X* **5**, 041017 (2015).

79. Z. Chen, X. Wang, S. Yu, Z. Li, H. Guo, Continuous-mode quantum key distribution with digital signal processing. *npj Quantum Inf.* **9**, 28 (2023).

80. T. Upadhyaya, “Tools for the security analysis of quantum key distribution in infinite dimensions,” thesis, University of Waterloo, Ontario, Canada (2021).
